# L2 growth mindset in AI-mediated language learning: effects of perceived usability and presence of generative AI chatbots

**DOI:** 10.3389/fpsyg.2025.1700117

**Published:** 2025-11-07

**Authors:** Yanbin Huang, Huanhui Chen, Changyue Hu

**Affiliations:** 1 Public Courses Department, Xiamen Medical College, Xiamen, Fujian, China; 2 Digital Intelligence Innovation and Entrepreneurship Industry College, Fuzhou University of International Studies and Trade, Fuzhou, Fujian, China

**Keywords:** perceived usability, presence, L2 growth mindset, emotional intelligence, willingness to communicate, AI-mediated language learning

## Abstract

**Introduction:**

This study examines perceived usability and presence of generative AI chatbots influence second language (L2) growth mindset in the context of AI-mediated oral learning.

**Methods:**

A SEM was tested with survey data from 530 Chinese university students engaged in AI-assisted oral English learning. The model examined direct and indirect relationships among perceived usability, presence, emotional intelligence, willingness to communicate (WTC), and L2 growth mindset.

**Results:**

Results show that perceived usability directly promotes L2 growth mindset and is indirectly influenced by WTC. Presence not only has a direct positive effect on L2 growth mindset but also has a dual positive mediating effect through emotional intelligence and WTC.

**Discussion:**

These findings suggest that AI chatbots act not only as learning tools but as cognitive partners, whose usability and immersive features foster learners’ belief in the malleability of language ability. The study advances theory on technology-supported growth mindset and provides empirical evidence to inform AI-driven language learning practices.

## 1 Introduction

The rapid adoption of generative AI in education is fundamentally transforming second language (L2) acquisition, especially in the area of speaking. AI conversational chatbots with natural language processing capabilities offer learners new possibilities for interactive speaking practice (Kusal et al., 2022; Razak et al., 2023). These instruments provide low-anxiety context, instant feedback, and personal interaction that have been found to mitigate foreign language anxiety and increase motivation for communication (Fathi et al., 2024; Tai and Chen, 2023). Yet beyond such proximate affective and behavioral effects, an important question is: In what ways do ongoing experiences with AI mould learners’ fundamental beliefs about their own learning potential? Central to this work is the idea of growth mindset—people’s belief that their abilities can be developed through effort and perseverance (Dweck, 2006; Tang et al., 2019).

Growth mindset is a strong predictor of motivation, engagement, stress, resilience, and self-efficacy in the learning of L2 (Derakhshan and Fathi, 2024; Jiang et al., 2024; Lee and Kwon, 2023; Lou and Noels, 2019; Yeager et al., 2022; Zhang and He, 2025). While social-contextual antecedents of mindset have been identified, such as teacher feedback, classroom autonomy, and parental support (Hejazi et al., 2023; Mesler et al., 2021; Yeager et al., 2022; Yu et al., 2022; Zarrinabadi et al., 2021; Zhang and He, 2025), there is still much to learn about the unique affordances offered by AI-mediated learning environments (Oruç, 2025; Zarrinabadi et al., 2021). These are the settings with high interactivity and presence (Shadiev et al., 2024), which introduce a new front line for mindset formation.

The Distributed Cognition Theory posits that cognition is not confined to an individual’s mind but is distributed across people, tools, and environments (Hollan et al., 2000). From this perspective, acting as a teacher, tutor, and collaborator in educational contexts (Fryer et al., 2019; Zapata-Rivera et al., 2024), a Generative AI chatbot is no longer a passive instrument but an active “symbiotic co-operator” in a dynamic human–machine cognitive system (Lee and Kwon, 2023; Peng and Liang, 2025). The learner and the AI mutually construct a cognitive system in which cognition is co-constructed in the interaction.

This conceptual lens uncovers a major discrepancy. It is still limited researched or theorized how AI chatbots are in symbiosis with humans within the framework of distributed cognition in an empirical manner (Peng and Liang, 2025), and what kind of tech (perceptions), such as perceived usability (the ease and effectiveness of use) and presence (as a sense of being with the AI), results in a growth mindset. This also echoes the insufficient exploration of the antecedents of language learning mindsets (Oruç, 2025; Zarrinabadi et al., 2021). Moreover, in technology-supported EFL learning, emotional intelligence (EI) mediates the relationship between digital tool use and academic performance by helping learners adapt, stay motivated, and engage socially (Alrajhi, 2024; Zhang J. et al., 2024). Willingness to communicate (WTC) is regarded as the immediate antecedent of actual communicative behavior and an important psychological variable for assessing learners’ investment in language learning (Lee and Taylor, 2024). EI and WTC via which such beliefs lead to more enduring beliefs are not obvious.

This gap is addressed in the current study, which explores how perceived usability and the presence of generative AI chatbots serve as contextual precedents for the L2 growth mindset, itself mediated by EI and WTC. Through this, it seeks to contribute to a theoretical understanding of technology-enhanced language learning while offering empirically based information for the development of AI-assisted pedagogical interventions.

## 2 Literature review

### 2.1 Growth mindset and second language learning

Growth mindset (GM), originating from Dweck’s theory of mind, emphasizes the belief that one’s abilities can be continuously developed through effort, effective strategies, and sustained learning (Dweck, 2006; Yeager et al., 2022). Early research focused on academic performance and gradually moved to non-cognitive domains such as emotion regulation, moral judgment, and creativity (Burnette et al., 2020).

In L2 learning, Lou and Noels (2017, 2019) constructed the Language Mindset Inventory and found that a growth language mindset is associated positively with learners’ goal orientations and adaptive reactions to difficulty. Empirical studies that investigate the predictors of growth mindset have mainly concentrated on social and personal influences. Socially, parents’ attitudes toward failure and their involvement contribute to children’s mindset development (He and Shi, 2025). Perceived teacher mindset, autonomy support, and supportive teacher–student relationships enhance students’ growth mindset (Li, 2023; Ma et al., 2020). Classroom opportunities for autonomy, peer interaction, and learning resources further promote it (Canning and Limeri, 2023; Zarrinabadi et al., 2021). At school level, a positive school climate strengthens the link between growth mindset and academic achievement (Zhang and He, 2025). Cross-cultural research also showed that learners are more likely to cultivate growth mindset in supportive environments (Canning and Limeri, 2023).

Personal factors such as students’ possible selves, use of metacognitive strategies, and sense of agency, foster confidence in their ability (Frazier et al., 2021). In addition, academic experiences and logical reasoning directly shape whether one tends to adopt a growth or fixed mindset (Limeri et al., 2020). EI also enables individuals to manage emotions and maintain healthy relationships, thereby equipping them to better cope with challenges in study and work (Katsumata and Teixeira, 2024).

One major drawback, however, is that these studies have generally been in standard or low-immersion online classrooms (Canning and Limeri, 2023), thus overlooking to a degree how high-interactivity AI-driven environments foster this belief.

### 2.2 Perceived usability and presence in AI-related contexts

#### 2.2.1 Perceived usability in AI-related contexts

Perceived usability of AI chatbots (PU) refers to the extent to which users perceive the system as effective and efficient in facilitating human–machine interaction, with emphasis on user satisfaction, ease of use, chatbot’s ability, conversational quality and privacy protection (Chauhan et al., 2024; Elliott and Soifer, 2022). According to Borsci et al. (2022), the perceived usability of AI chatbots incorporates five dimensions: perceived accessibility, functional quality, dialogue quality, privacy and security, and response time.

Perceived usability is a key factor influencing EFL learners’ acceptance and use of technology-assisted tools (Chauhan et al., 2024). Many studies show it predicts learners’ attitudes, intentions, and actual use of digital resources (Khan et al., 2021; Liu, 2023; Rafiee and Abbasian-Naghneh, 2019). When interfaces are easy to navigate, learners are more engaged, focused, motivated and report greater course satisfaction (Ebadi and Raygan, 2023; Liu and Ma, 2023; Lu et al., 2022), and well-designed privacy safeguards and transparent security measures foster acceptance and greater engagement (Mutimukwe et al., 2022; Zhang, 2024), all of which are fundamental to continued effort—a central aspect of growth mindset.

#### 2.2.2 Presence in AI-related contexts

Presence (PR) refers to the degree to which users experience a sense of “being there” in the context of virtual reality and computer-mediated communication (van Brakel et al., 2022). It arises not only from the immersive features but also from the combined effects of social interaction, cognitive processing, and emotional projection. In the educational context, presence is conceptualized as teaching, cognitive, and social presence (Debets et al., 2025; Garrison and Arbaugh, 2007). Among them, social presence is critical, as it reflects learners’ awareness of others and their ability to build emotional connections and trust (Debets et al., 2025; Li et al., 2019).

High levels of presence have a positive influence on learners’ cognitive, affective, and behavioral outcomes. Research shows that stronger presence enhance motivation, engagement, and interaction (Hew et al., 2023; Swan and Shih, 2019; Zuo et al., 2022). Presence strengthens social connectedness by offering emotional support that fosters positive emotions, memory, and resilience, especially in tasks requiring sustained effort (Makowski et al., 2017). AI chatbots equipped with social response features and multimodal resources such as voice, text, and avatars responding empathetically and coherently in chat can lead to high presence, which increase learners’ sense of participation and emotional engagement (Busso and Sanchez, 2024; Wan and Moorhouse, 2024; Wang C. et al., 2024; Zou et al., 2023), enhance their trust and willingness to use the technology (Hite et al., 2019), and enhance concentration, comprehension, and knowledge retention (Hite et al., 2019; Huang et al., 2019). These features can truly help learning feel more real and give the confidence that they are really improving.

Although perceived usability and presence have been widely examined in technology-enhanced learning, existing studies have primarily focused on their effects on learners’ motivation, satisfaction, and continued intention to use technology. However, little is known about how these factors may influence deeper psychological traits such as growth mindset. Understanding whether and how perceived usability and presence shape learners’ growth mindset can reveal the underlying psychological mechanisms through which AI-mediated learning environments exert their influence.

### 2.3 Emotional intelligence

Emotional Intelligence (EI) is the ability to perceive, understand, manage, and use emotions (Salovey and Mayer, 1990). It has become not only an important construct in psychology but also a central variable of interest in education (Elfenbein and MacCann, 2017). Several theoretical models of EI have been proposed, with trait EI and ability EI being the most widely used. Trait EI, typically measured through self-report instruments, emphasizes individuals’ self-perceptions of their emotional capabilities, whereas ability EI is assessed through performance-based tests that evaluate actual skills in processing and responding to emotions (Kopp et al., 2021). The most popular trait EI framework is self-emotion appraisal, others’ emotion appraisal, self-emotion regulation, others’ emotion regulation, and the use of emotions (Davies et al., 2010).

In L2 contexts, EI is a crucial psychological variable to shape learners’ emotional and communicative experiences. Many empirical studies show that high EI is significantly associated with lower foreign language anxiety and communication apprehension, as emotionally intelligent learners regulate their emotions more effectively during oral tasks (Cong and Li, 2022; Han et al., 2022). They demonstrate greater self-confidence and stronger WTC, leading to more active participation and sustained engagement (Gao et al., 2025; Alamer and Alrabai, 2024; Shao et al., 2025). Among Chinese university EFL learners, trait EI further buffers against boredom and academic burnout, helping sustain motivation and improve English learning performance (Zhang T. et al., 2024).

In technology-supported EFL learning, EI mediates the relationship between digital tool use and academic performance. Learners with high emotional intelligence are better able to adapt to new platforms, maintain learning motivation, and effectively cope with learning challenges (Zhang J. et al., 2024). This highlights EI’s dual role in regulating emotions and sustaining long-term language mindsets across diverse instructional contexts.

In sum, EI not only influences learners’ emotions, engagement, and performance but may also mediate the relationship between external perceptions (e.g., usability, presence) and L2 speaking outcomes. Yet, how EI performs this mediating role in chatbot-supported environments remains insufficiently understood, pointing to an important direction for future research.

### 2.4 Willingness to communicate

Willingness to communicate (WTC) is commonly defined as learners’ readiness to initiate communication in the L2 with a specific person at a particular time (MacIntyre et al., 1998). Given the focus of this study on AI-mediated environments, WTC is conceptualized here as learners’ inclination to engage in interaction with AI using the target language (Peng and Liang, 2025).

Traditional research on the antecedents of WTC has primarily emphasized positive psychology and contextual factors. Research has shown that WTC is closely associated with factors such as enjoyment, grit, growth language mindset, and ideal L2 self, with growth mindset serving as a key mediating pathway (Fathi et al., 2024; Kim and Su, 2024; Lee and Taylor, 2024; Tai and Chen, 2023; Wang C. et al., 2024; Zarrinabadi et al., 2021). Besides that, emotional factors are also important. Positive emotions and supportive classroom climates enhance WTC, whereas anxiety and boredom undermine it. In addition, contextual factors such as teacher support and classroom climate also play important roles in influencing WTC, especially on students with higher growth mindset (Hejazi et al., 2023). These findings indicate that WTC is not a single linguistic performance variable, but also a reflection of learners’ motivation, emotions, and language mindsets.

In technology-supported EFL learning contexts, AI chatbots have also been shown to enhance learners’ WTC (Tai and Chen, 2023; Wang C. et al., 2024; Zhang and Ma, 2024). While traditional factors influencing WTC remain relevant in WTC with AI contexts, AI-specific variables such as perceived ease of use, perceived usefulness, and attitudes toward use play an even more critical role (Peng and Liang, 2025). Longitudinal findings further suggest that learners experienced a sense of human-like interaction during sustained exchanges with AI forms “anthropomorphic perception” to facilitate higher WTC with AI (Peng and Liang, 2025). Yet, most existing studies emphasize language outcomes or affective variable (e.g., anxiety, enjoyment, motivation), with limited attention to AI-specific factors or the mechanisms through which they shape WTC and its relationship with growth mindset.

In summary, existing studies have, respectively, revealed the roles of perceived usability and presence in technology adoption and learning experience, the crucial function of emotional intelligence in emotion regulation and learning engagement, and the central position of WTC in L2 learning behaviors. Research on growth mindset has also highlighted its significance in shaping learners’ motivation, effort, and resilience in language learning. However, these factors have not yet been integrated to explain how L2 growth mindset develops in AI-mediated contexts.

Accordingly, this study addresses the following research questions:

RQ1: Do learners’ perceptions of AI chatbots’ usability and presence directly influence their L2 growth mindset?

RQ2: Do emotional intelligence and willingness to communicate with AI mediate perceived usability and presence and L2 growth mindset?

## 3 Model and hypothesis

Distributed cognition emphasizes that technology is no longer a passive tool but an active “collaborator” within the cognitive system, where learners and AI together form a dynamic cognition–interaction partner. Cognitive activity does not reside solely in the individual mind but is distributed across the interconnected system of people, tools, and environments. In this view, cognition is accomplished through the coordinated functioning of these elements, with external technologies shaping cognitive processes in a participatory manner (Hollan et al., 2000).

Based on this perspective, this study hypothesizes that the AI chatbot is a cognitive partner. The external technical elements (usability, presence) are incorporated into the learner’s cognitive structure to affect internal psychological states (EI, WTC), which in turn shape the metacognitive belief of growth mindset. The specific research hypotheses are outlined as follows:

Under the distributed cognition framework, learners’ cognitive processes are collaboratively accomplished through the interaction of individuals, tools, and environmental elements (Grinschgl and Neubauer, 2022), so external technologies participate in shaping cognition, and cognitive load during learning can be shared or distributed across external resources.

AI chatbots provide EFL students with a stable and easily accessible platform for oral practice, offering diverse scenarios and high-quality feedback, delivering guiding feedback, structuring tasks, and scaffolding learning that reduces cognitive load, enhances motivation and satisfaction, and encourages learners to practice more frequently, follow guidance, and learn from mistakes. In doing so, they foster greater engagement and help cultivate resilience in learning (Chen et al., 2024). Through these interactions, learners engage in reflection and meaning-making, which increases their participation in knowledge construction and strengthens their beliefs in growth mindset.

AI chatbots can support learners in using language appropriately across different contexts, managing turn-taking effectively, and improving discourse organization. Through sustained interaction with chatbots, learners are able to practice and refine compensatory and interactional strategies (Huang et al., 2024; Wang C. et al., 2024), thereby strengthening their emotional intelligence.

Moreover, the non-judgmental nature of chatbot interaction significantly reduces communicative pressure and anxiety in the process of foreign language learning. By creating a safe and inclusive communicative environment and offering encouraging feedback, chatbots motivate learners to take risks, practice repeatedly, and express themselves more freely (Fathi et al., 2024; Tai and Chen, 2023; Wang C. et al., 2024; Zhang and Ma, 2024), which in turn enhances their WTC.

Based on these arguments, the following hypotheses are proposed:

H1a: Perceived usability has a significant positive effect on learners’ L2 growth mindset.

H1b: Perceived usability has a significant positive effect on learners’ emotional intelligence.

H1c:Perceived usability has a significant positive effect on learners’ WTC.

Within the framework of distributed cognition, presence serves as an essential attribute of human–machine collaboration. Presence strengthens social connectedness by providing emotional support, which in turn fosters positive emotions, enhances memory, and builds learners’ psychological resilience (Makowski et al., 2017), thereby facilitating the development of a growth mindset.

By leveraging multimodal resources such as voice, text, and avatars, AI systems can create immersive, high-presence learning experiences that heighten learners’ sense of participation and emotional engagement (Busso and Sanchez, 2024; Wan and Moorhouse, 2024; Wang C. et al., 2024).

Generative AI is also capable of flexibly adopting multiple roles, as teacher, collaborator, or peer, and through affective responses, guiding prompts, and personalized task design, it provides continuous social and cognitive support (Fryer et al., 2019; Yang et al., 2022; Yang et al., 2024). Such supportive environments foster positive emotional experiences and foreign language enjoyment, thereby enhancing WTC (Huang et al., 2024; Lee and Kwon, 2023).

Based on these considerations, the following hypotheses are proposed:

H2a: Perceived presence of generative AI chatbots has a significant positive effect on learners’ L2 growth mindset.

H2b: Perceived presence of generative AI chatbots has a significant positive effect on learners’ emotional intelligence.

H2c: Perceived presence of generative AI chatbots has a significant positive effect on learners’ WTC.

Within the framework of distributed cognition, emotion intelligence is not solely dependent on individuals’ internal capacities but is partially externalized and distributed through human–AI collaboration. As a “collaborator” in the cognitive system, AI chatbots can provide external resources for learners’ emotional regulation through non-judgmental responses, affective feedback, and social support functions (Kim and Su, 2024; Rezai et al., 2024; Wang C. et al., 2024).

Research has shown that learners with higher levels of emotional intelligence are better able to perceive both their own and others’ emotions (Elfenbein and MacCann, 2017; Ye et al., 2024). Those with stronger abilities in emotion perception and regulation are more capable of overcoming difficulties (Xing et al., 2023), which aligns with the development of a growth mindset.

Based on this reasoning, the following hypothesis is proposed:

H3a: Emotional intelligence has a significant positive effect on L2 growth mindset.

Perceived usability and presence, as experiential variables of external technologies, can act as collaborators in shaping cognitive processes (Peng and Liang, 2025). Both technological usability and social presence directly strengthen learners’ growth mindset by enhancing their sense of control, immersion, and social interaction (Zarrinabadi et al., 2021). However, the transmission of these external experiences into internal beliefs often requires learners’ emotional processing capacity as a psychological bridge (Seitz, 2022). In the human–AI collaborative distributed system, learners’ emotional intelligence enables them to perceive and regulate their emotions more effectively. Learners with higher levels of emotional intelligence are better able to transform the positive emotional experiences brought about by usability and presence into growth mindset and sustained motivation for effort.

Based on this reasoning, the following hypotheses are proposed:

H3b: Emotional intelligence mediates the relationship between perceived usability and L2 growth mindset.

H3c: Emotional intelligence mediates the relationship between perceived presence and L2 growth mindset.

From the perspectives of distributed cognition, WTC is a dynamically generated outcome of interaction within the human–AI collaborative system. Frequent interaction with a “cognitive partner,” such as an AI system, enhances learners’ WTC, providing immediate feedback and repeated successful experiences (Peng and Liang, 2025). In communicative exchanges, learners also exercise agency, attributing their progress to controllable factors, which reinforces the belief that “ability can be improved through effort” and thus promotes a growth mindset (Zarrinabadi et al., 2021).

Accordingly, the following hypothesis is proposed:

H4a: WTC has a significant positive effect on learners’ growth mindset.

In the context of language learning, when learners perceive AI-based platforms as effective in improving language proficiency and providing valuable support, they are more likely to actively engage in communicative activities (Peng and Liang, 2025). The WTC inspired by interaction with AI encourages learners to use external resources more actively and engage in communication within the distributed system. The WTC fostered through AI interaction offers more opportunities to receive feedback and accumulate mastery experiences (Evenddy, 2024), thereby reinforcing growth mindset.

Thus, the following hypotheses are proposed:

H4b: WTC mediates the relationship between perceived usability and L2 growth mindset.

H4c: WTC mediates the relationship between perceived presence and L2 growth mindset.

**Figure 1:**
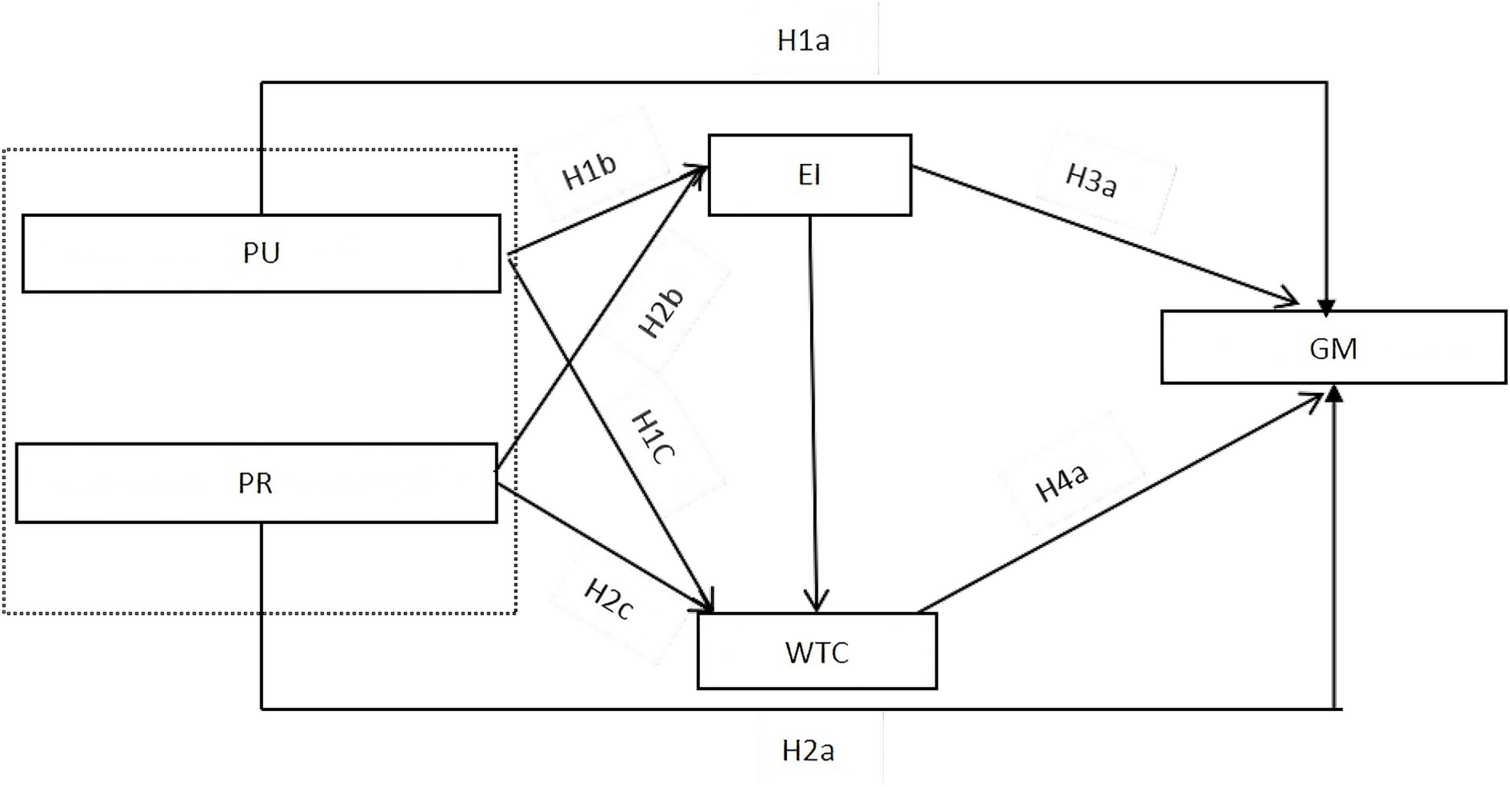
Conceptual model.

Based on the above analysis and hypotheses, the conceptual model of this study is illustrated in Figure 1.

## 4 Method

### 4.1 Participants and data collection

This research was carried out at four undergraduate universities in South China. To be included in the study, participants had to be non-native English speakers who had studied English for at least 3 years, were currently in college and taking an English course, and importantly, indicated that they had used generative AI speaking assistants for practicing speaking. In the pre-test questionnaire, participants were asked to report the main AI tools they frequently used, such as ChatGPT and AI Cicibot. These tools included both general interaction platforms based on large language models (e.g., ChatGPT) and specialized chatbots designed for speaking practice (e.g., Cicibot).

Data were collected between April 20 and May 20, 2025. The questionnaire was uploaded to the Wenjuanxing platform and distributed through QQ groups with random sampling via QR code. Prior to completing the survey, participants were presented with an informed consent form and screening questions about their English learning background and use of generative AI chatbot. The study received approval from the Institutional Ethical Review Board of the researchers’ institution. A total of 530 valid questionnaires were kept for analysis following data cleansing after an initial response rate of 592.

### 4.2 Instrument

The questionnaire instrument consisted of two sections. The first section collected demographic information, including participants’ age, gender, grade, English proficiency level, and their experience of AI use. The second section comprised five subscales: perceived usability (15 items), presence (8 items), L2 growth mindset (9 items), emotional intelligence (10 items), and WTC (5 items). All constructs were assessed on 5-point modified previously validated scales except for L2 Growth Mindset (6-point scale) and were measured on a 5-point Likert scale (1 = Strongly Disagree to 5 = Strongly Agree).

#### 4.2.1 Perceived usability of AI chatbots (PU)

This construct was measured using the *Chatbot Usability Scale* developed by Borsci et al. (2022). The instrument was originally designed to assess user interaction with AI conversational agents and closely aligns with the interactive features in this study. The scale consists of five dimensions: perceived accessibility, functional quality, dialogue and information quality, privacy and security, and response time. To better fit the context of AI-assisted oral learning, the original wording was adapted by replacing references to “general scenarios” with “oral learning scenarios,” ensuring that the items captured learners’ perceptions of chatbot usability.

#### 4.2.2 Presence of AI chatbots (PR)

Presence of AI chatbots was measured by an adapted version of Law’s Presence Scale (Law et al., 2019), which has been widely applied in technology-mediated distance education. The scale comprises three sub-dimensions: social presence, cognitive presence, and teaching presence. To align with the purpose of this study, the target of perception was modified from “educational technology” to “AI chatbot,” making the instrument suitable for the human–AI oral interaction context.

#### 4.2.3 L2 growth mindset (GM)

It was assessed using the Language Mindset Inventory developed by Lou and Noels (2017). The original instrument consists of three dimensions: mindset about general language intelligence, attitude, and age sensitivity, with both growth- and fixed-mindset items under each dimension. Since the present study specifically focuses on growth-oriented beliefs, nine items from the dimensions of L2 growth language mindset were chosen.

#### 4.2.4 Emotional intelligence (EI)

Learners’ emotional intelligence was measured using the Brief Emotional Intelligence Scale (BEIS) developed by Davies et al. (2010). The instrument consists of five dimensions: self and others’ emotional appraisal, self and other emotion regulation, and utilization of emotion.

#### 4.2.5 Willingness to communicate with AI (WTC)

Learners’ WTC with AI was assessed using the WTC scale originally developed by Tai and Chen (2023). The original instrument was designed to measure students’ WTC with Google Assistant; in the present study, the term *Google Assistant* was replaced with *AI conversational chatbot* to fit the research context.

### 4.3 Data analysis

Data were analyzed using SPSS 26.0 and AMOS 23.0. First, all questionnaire items were coded and transformed according to the scale metrics, followed by descriptive statistics and correlation analyses. Second, confirmatory factor analysis (CFA) was conducted to examine the discriminant validity of the constructs and to assess potential common method bias, as well as the overall model fit. Finally, structural equation modeling (SEM) was employed to test the hypothesized relationships, with the bootstrapping method applied to evaluate the mediating effects.

## 5 Results

### 5.1 Descriptive statistic analysis

The descriptive statistics of the sample show that participants were aged between 18 and 25 years, with an average age of 19.64 (SD = 1.38). Among them, 181 were male (34.2%) and 349 were female (65.8%). Regarding English proficiency, 137 participants (25.8%) reported below CET-4 level, 306 (57.7%) at CET-4 level, 69 (13.0%) at CET-6 level, and 18 (3.4%) above CET-6 level, which is indicative of the target Chinese university EFL population. See Table 1.

**Table 1:** Demographic characteristics of research sample (*N* = 530).

Variable	Frequency	%
Gender		
Male	181	34.2
Female	349	65.8
Age		
18	108	20.4
19	174	32.8
20	139	26.2
≥21	109	21.6
Grade		
First	267	50.4
Second	165	31.1
Third	66	12.5
Fourth	29	5.5
Fifth	3	0.6
English proficiency		
B1-below CET4	137	25.8
B2-CET4	306	57.7
B3-CET6	69	13.0
B4-above CET6	18	3.4

### 5.2 Reliability and validity

To examine the stability and internal consistency of the scales, this study used SPSS 23.0 to calculate Cronbach’s *α* coefficients for each latent variable. The results showed that PU was 0.933, PR was 0.908, GM was 0.869, EI was 0.877, and WTC with AI was 0.880. All values exceeded the threshold of 0.70, indicating good internal consistency, high overall reliability, and satisfactory measurement stability.

The factor loadings of PU, PR, GM, EI, and WTC with AI ranged from 0.558 to 0.920. The composite reliability (CR) values for these constructs were 0.874, 0.906, 0.786, 0.854, and 0.882, all exceeding the recommended threshold of 0.70. The average variance extracted (AVE) values for PU, PR, GM, EI, and WTC with AI were 0.590, 0.763, 0.554, 0.540 and 0.600, respectively, all greater than 0.50. These results indicate that the constructs demonstrated good convergent validity (Fornell and Larcker, 1981).

The Kaiser-Meyer-Olkin (KMO) value of the questionnaire was 0.953, exceeding the recommended threshold of 0.70. Bartlett’s test of sphericity was significant (*p* = 0.000), indicating good content validity and suitability for factor analysis. The results of Harman’s single-factor test showed that the variance explained by the first factor was 35.73%, which is below the critical value of 40%, suggesting that there was no serious common method bias in this study. Details are presented in Table 2.

**Table 2:** Statistical outcomes of confirmatory factor analysis.

Variable	Loading (>0.5)	Cronbach’s *α* (>0.7)	CR (>0.7)	AVE (>0.5)	Eigenvalues	
					Total	% of Variance
PU		0.933	0.874	0.590	16.793	35.729
PU1	0.653					
PU2	0.920					
PU3	0.910					
PU4	0.558					
PU5	0.735					
PR		0.908	0.906	0.763	3.463	7.367
PR1	0.851					
PR2	0.904					
PR3	0.864					
GM		0.869	0.786	0.554	2.529	5.381
GM1	0.803					
GM2	0.805					
GM3	0.608					
EI		0.877	0.854	0.540	1.988	4.229
EI1	0.725					
EI2	0.743					
EI3	0.784					
EI4	0.647					
EI5	0.768					
WTC		0.880	0.882	0.600	1.526	3.248
WTC1	0.808					
WTC2	0.815					
WTC3	0.704					
WTC4	0.719					
WTC5	0.818					
KMO 0.953 Bartlett’s Test Sig *p* = 0.000						

CR, construct reliability; AVE, average variance extracted.

For PU, PR L2 GM, EI, and WTC with AI, the square roots of the Average Variance Extracted (AVE) were 0.768, 0.871, 0.743, 0.734, and 0.774, respectively. All values were greater than the corresponding correlations with other variables, indicating good discriminant validity. See Table 3.

**Table 3:** Discriminant validity for the measurement model.

Construct	PU	PR	GM	EI	WTC
PU	**0.768**				
PR	0.741**	**0.871**			
GM	0.541**	0.532**	**0.743**		
EI	0.431**	0.468**	0.435**	**0.734**	
WTC	0.615**	0.640**	0.541**	0.534**	**0.774**

The items on the diagonal represent the square roots of the AVE; off-diagonal elements are the correlation estimates; **Correlation is significant at the level 0.01 level (2-tailed).

### 5.3 Model fit test

The SEM demonstrated a good fit to the data: *χ*^2^(179) = 378.94, *p* < 0.001, *χ*^2^/df = 2.12. The goodness-of-fit indices were satisfactory, with CFI = 0.970, TLI = 0.965, NFI = 0.945, GFI = 0.934, RMSEA = 0.046, SRMR = 0.034. Overall, these results suggest that the hypothesized model fits the data well (Wu, 2013).

To further validate the goodness of fit of the proposed theoretical model, this study conducted a nested model comparison with two competing models: a higher-order model (full mediation model) and a lower-order model (direct effects model). The results showed that the theoretical model demonstrated significantly better fit than the higher-order model (Δ*χ*^2^ = 39.64, Δdf = 2, *p* < 0.001). Similarly, when compared with the lower-order model, the theoretical model also exhibited a significant advantage (Δ*χ*^2^ = 561.42, Δdf = 7, *p* < 0.001). Therefore, the proposed theoretical model was superior to the competing models and provided a better explanation of the data. Details are presented in Table 4.

**Table 4:** Model comparison.

Model fit indices	Proposed model	Higher-order model	Lower-order model
*χ* ^2^	378.94	418.576	940.361
df	179	181	186
*χ*^2^/df	2.12	2.313	5.056
CFI	0.970	0.965	0.887
TLI	0.965	0.959	0.873
RMSEA	0.046	0.050	0.088
SRMR	0.0335	0.0442	0.2788
Δ*χ*^2^ vs. higher-order model	39.64		
Δdf vs. higher-order model	2		
*p* value vs. higher-order model	<0.001		
Δ*χ*^2^ vs. lower-order model	561.42		
Δdf vs. lower-order model	7		
*p* value vs. lower-order model	<0.001		

### 5.4 Structural equation model test

**Figure 2:**
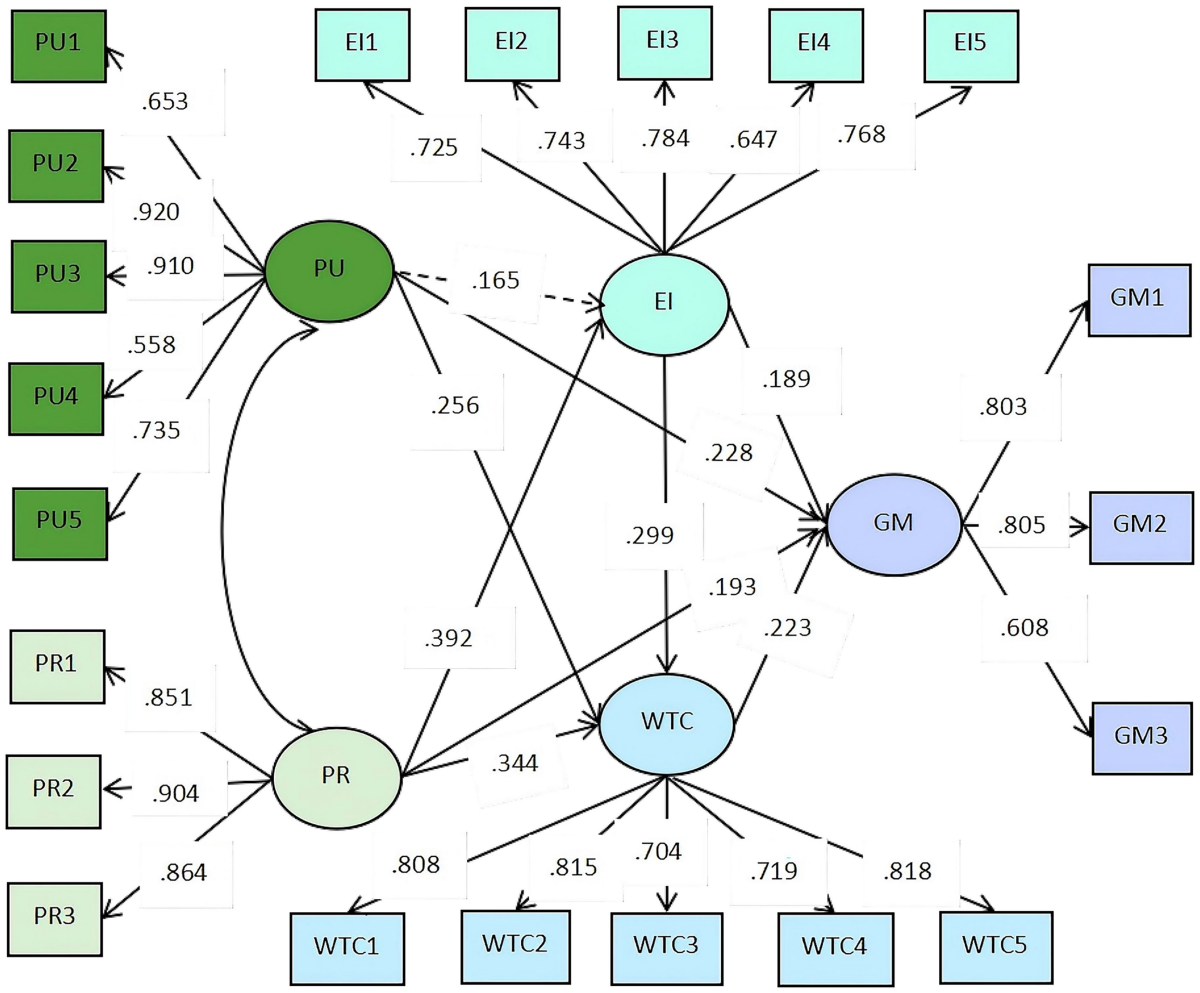
Structural modal for growth mindset. PU, perceived usability; PR, perceived presence; EI, emotional intelligence; WTC, willingness to communicate with AI; GM, second language growth mindset. The coefficients shown are standardized path coefficients. Statistically significant paths were presented by solid lines and insignificant path was presented by dotted path.

The results of the tested model with standardized path coefficients are presented in Figure 2. Results indicated that PU had a significant positive effect on GM (H1a: *β* = 0.648, S.E. = 0.234, C.R. = 2.767, *p* = 0.006), supporting the hypothesis. However, the effect of PU on EI was not significant (H1b: *β* = 0.279, S.E. = 0.149, C.R. = 1.875, *p* = 0.061), and thus the hypothesis was not supported. In contrast, PU significantly predicted learners’ WTC (H1c: *β* = 0.277, S.E. = 0.076, C.R. = 3.639, *p* < 0.001), providing support for the hypothesis.

PR was found to exert significant positive effects on GM (H2a: *β* = 0.239, S.E. = 0.108, C.R. = 2.202, *p* = 0.028), EI (H2b: β = 0.288, S.E. = 0.066, C.R. = 4.352, *p* < 0.001), and WTC (H2c: *β* = 0.162, S.E. = 0.034, C.R. = 4.687, *p* < 0.001), thus supporting all three hypotheses.

Furthermore, both EI and WTC significant positive effects on GM (H3a: *β* = 0.318, S.E. = 0.094, C.R. = 3.393, *p* < 0.001; H4a: *β* = 0.586, S.E. = 0.186, C.R. = 3.146, *p* = 0.002). Detailed results are presented in Table 5.

**Table 5:** Path coefficients and the hypothesis testing results.

H+Path	*β*	S.E.	C.R.	*p*	Result
H1a: PU → GM	0.648	0.234	2.767	0.006	Support
H1b: PU → EI	0.279	0.149	1.875	0.061	Reject
H1c: PU → WTC	0.277	0.076	3.639	<0.001	Support
H2a: PR → GM	0.239	0.108	2.202	0.028	Support
H2b: PR → EI	0.288	0.066	4.352	<0.001	Support
H2c: PR → WTC	0.162	0.034	4.687	<0.001	Support
H3a: EI → GM	0.318	0.094	3.393	<0.001	Support
H4a: WTC → GM	0.586	0.186	3.146	0.002	Support

The results of the bias-corrected bootstrap test of mediation effects revealed that the indirect effect of PU on GM through EI [H3b: *β* = 0.088, 95% BC CI (−0.013, 0.336), *p* = 0.107] was not statistically significant. However, the indirect effect of PU on GM through WTC [H4b: *β* = 0.166, 95% BC CI (0.026, 0.430), *p* = 0.012] reached a significant level.

The indirect effects of PR on GM were both significant through EI [H3c: *β* = 0.089, 95% BC CI (0.027, 0.205), *p* = 0.009] and WTC [H4c: *β* = 0.096, 95% BC CI (0.033, 0.201), *p* = 0.005], with the confidence intervals not containing zero. Detailed results are shown in Table 6.

**Table 6:** Indirect effect testing results.

H+Path	*β*	95% BC	*p*	Result
H3b: PU → EI → GM	0.088	[−0.013, 0.336]	0.107	Reject
H3c: PR → EI → GM	0.089	[0.027, 0.205]	0.009	Support
H4b: PU → WTC → GM	0.166	[0.026, 0.430]	0.012	Support
H4c: PR → WTC → GM	0.096	[0.033, 0.201]	0.005	Support

## 6 Conclusion and discussion

### 6.1 Conclusion

This study aims to investigate how technological perceptions, as contextual antecedents, influence L2 growth mindset in AI-mediated oral learning contexts. By constructing a structural equation model, it examines the direct and indirect pathways through which perceived usability and presence of AI chatbots affect growth mindset, and further verifies the mediating roles of emotional intelligence and WTC. The findings thus reveal a chain mechanism linking technological interaction, emotional willingness, and mindset construction, offering a theoretical contribution to explaining the psychological mechanisms underlying AI-supported language learning.

The findings indicate that perceived usability has a significant direct positive effect on growth mindset. This result resonates with prior research emphasizing that highly usable platforms can reduce learners’ cognitive load, encourage them to engage in frequent practice, accept feedback, and learn from errors, thereby fostering learning resilience, which is an essential element of Dweck’s conceptualization of growth mindset (Chen et al., 2024; Dweck, 2006). Further analysis revealed that perceived usability not only directly influences growth mindset but also indirectly contributes to it through learners’ WTC with AI. This finding corroborates Peng and Liang’s argument that learners and artificial intelligence tools form a “cognitive community,” in which the more usable and accessible the technology is perceived to be, the stronger the learners’ WTC with it (Peng and Liang, 2025). In communicative interactions, AI chatbots act as interlocutors by providing learners with immediate and level-adaptive feedback, constructing scaffolding for language development, and enabling learners to accumulate successful experiences through repeated human–AI exchanges. Such experiential accumulation facilitates the development of a growth mindset and further corroborates the finding that peer support contributes to the formation of growth-oriented beliefs (Namaziandost et al., 2024). Moreover, within the cognitively co-constructed community formed between learners and AI, learners take the initiative in communication. This sense of agency further contributes to the development of a growth mindset, echoing Zarrinabadi et al.’s (2021) findings that autonomy-supportive students are more likely to endorse growth-oriented beliefs and, consequently, perceive themselves as more competent.

This study also revealed that presence has a significant direct effect on growth mindset. At its core, growth mindset represents a metacognitive belief about the malleability of language learning ability, a belief that often develops through the accumulation of cognitive experiences (Dweck, 2006). From the perspective of distributed cognition, such a belief does not stem from the individual alone but emerges from the interplay of personal cognition and human–AI interaction (Peng and Liang, 2025). AI chatbots, with their coherence and contextual simulation capabilities, situate learners in immersive, quasi-authentic language practice environments. Prior research has shown that immersion of this kind can substantially enhance engagement, knowledge retention, and overall learning outcomes (Canning and Limeri, 2023). The visible progress achieved in such contexts provides learners with tangible evidence that language ability can be improved through sustained practice. Over time, this evidence is internalized as a growth-oriented mindset. As Jonassen and Rohrer-Murphy (1999) argued, technology, when appropriated as a tool for active meaning-making, does not merely extend cognition but fosters the kind of intellectual skills that are enacted rather than possessed.

Presence not only has a direct positive effect on growth mindset but also has indirect effects through the dual pathways of emotional intelligence and WTC, unveiling a more interactive and emotionally engaging mechanism of psychological construction. High presence can stimulate learners’ emotional awareness and regulation abilities, enabling them to more effectively identify and manage language anxiety during interactions with AI chatbots, thereby fostering a more positive learning mindset and a stronger sense of self-efficacy. This finding resonates with earlier study that AI chatbots can assist learners in developing skills such as emotion regulation and stress management (Tripathi and Jyotsana, 2024). When learners perceive strong social presence, they are more likely to regard the AI as an interactive partner, thereby enhancing their motivation and willingness to communicate. Such positive interaction experiences encourage learners to continuously practice their language use during conversations, which in turn fosters their belief in the malleability of language learning. This finding proved the previous study that social presence enhances users’ perception of the chatbot’s “human-like” qualities and interactive capacity, thereby strengthening their willingness to engage in communication (Zhang J. et al., 2024).

The findings of this study also reveal a two-way relationship between growth mindset, WTC, and emotional intelligence. This is different from previous research. Prior research has largely emphasized that a growth mindset positively predicts learners’ WTC and emotional intelligence (Ebn-Abbasi et al., 2024; Hejazi et al., 2023; Wang H. et al., 2024; Xing et al., 2023; Xu and Zhang, 2025). The present results demonstrate a reverse effect as well: both WTC with AI and emotional intelligence can foster the growth mindset in turn. This suggests that the relationship between them is not a simple one-way but rather a dynamic and reciprocal mechanism. The reason may lie that from a distributed cognition view, cognition is “coupled” with tools, tasks, and social partners (Hollan et al., 2000); frequent and low-risk interaction with AI chatbot, viewed as partner thus sustains a positive feedback loop linking external conditions, like immediate scaffolding and internal beliefs, helping to grow a mindset that, in turn, further amplifies WTC and emotion regulation.

Finally, the study also shows that the perceived usability of AI does not have a significant direct effect on emotional intelligence. This may suggest that only recognition of a tool’s practicality is not sufficient to directly enhance one’s emotional awareness or regulation. Instead, the development of emotional intelligence appears to rely more heavily on authentic social interaction and sustained emotional training (Polonio-López et al., 2019). In other words, while AI tools can indeed trigger certain emotional responses, the effective cultivation of emotional intelligence still calls for a broader range of strategies, such as contextualized dialogues.

In conclusion, this study reveals that perceived usability and presence not only have direct effects on learners’ growth mindset but also operate indirectly through emotional intelligence and WTC. These findings indicate that the affordances of generative AI extend beyond serving as a simple learning tool; they function as important contextual factors that shape learners’ beliefs and psychological development.

### 6.2 Implication

#### 6.2.1 Theoretical implications

The findings of this study offer theoretical contributions by providing a perspective on the application of AI chatbot in the cultivation of students’ growth mindset. Grounded in the perspective of distributed cognition, it emphasizes that learners’ cognitive resources, emotional experiences, and metacognitive regulation are not confined to the individual but are extended and reorganized through human–AI collaboration within the external environment, thereby enriching the contextual antecedent analysis of growth mindset. This provides a theoretical supplement for understanding the psychological mechanisms of AI-supported language learning. Moreover, the study reveals that WTC with AI and emotional intelligence also has reciprocal effects on growth mindset. This indicates that the relationship among growth mindset, WTC, and emotional intelligence is not a one-way causal link but rather a dynamic, interactive mechanism, which deepens our understanding of growth mindset.

#### 6.2.2 Practical implications

From a practical perspective, this study offers implications for both AI tool developers and language educators.

For AI tool developers, first, the design of AI-mediated language learning tools should prioritize the optimization of interaction interfaces and task logic, ensuring efficiency and stability in system response, speech recognition accuracy, and task navigation. More importantly, such tools should provide visualized feedback on progress, as well as scaffolded support at different levels to enhance users’ perceived usability. In addition, AI developers can embed emotion-sensing and empathetic feedback mechanisms into the system, enabling learners to perceive and respond to emotions during interaction, thereby fostering more positive learning experiences. Furthermore, developers can create a human-like, emotionally responsive environment through multimodal design and the maintenance of a consistent AI persona, which enhances learners’ sense of social presence and, in turn, stimulates their emotional intelligence, willingness to communicate, and growth mindset.

For language educators, since perceived usability and presence have significant direct effects on learners’ growth mindset, first, it is essential to emphasize the construction of learners’ perceptions of usability and presence in class. When organizing AI-mediated speaking tasks, teachers should keep the learning process clear and coherent, with transparent feedback mechanisms that help students recognize their learning goals and track their progress. By providing timely feedback and visual evidence of improvement, educators can help students experience the connection between effort and progress, thereby strengthening their belief in development. In addition, since emotional intelligence and willingness to communicate play mediating roles in AI-assisted speaking learning, teachers are encouraged to integrate emotion-awareness activities and reflective dialogue into their task design. Guiding students to notice and reflect on their emotional responses during interactions with AI can enhance their ability to regulate emotions and maintain a constructive learning attitude. Creating a psychologically safe and supportive environment further helps reduce anxiety and increase confidence and willingness to communicate, ultimately fostering the parallel development of students’ language proficiency and growth mindset.

### 6.3 Limitations and future research

Although this study yields insightful findings regarding the relationships among perceived usability, presence, emotional intelligence, WTC, and growth mindset, there are still several limitations.

First, the possibility of making definitive causal inferences was limited by the cross-sectional nature of the study as a first limitation. Further studies on causality should use either longitudinal or experimental designs.

Second, the participants were limited to university students. Their language learning experience, frequency of tool use, and cultural background may restrict the external validity of the findings. To improve generalizability, future research could expand the sample to include learners across different age groups, linguistic backgrounds, and educational stages.

Third, although this study included key mediators, other factors such as learning motivation and self-efficacy may also moderate these relationships and should be included in more complex models.

## Data Availability

The raw data supporting the conclusions of this article will be made available by the authors, without undue reservation.
